# Cognition errors in the treatment course of patients with anastomotic failure after colorectal resection

**DOI:** 10.1186/s13037-019-0184-6

**Published:** 2019-01-23

**Authors:** P. Vogel, D. H. V. Vogel

**Affiliations:** 1 Abt. Allgemein-Viszeral- und Minimalinvasive Chirurgie, Klinikum Bad Hersfeld, Seilerweg 29, 36251 Bad Hersfeld, Germany; 2 Klinik und Poliklinik für Chirurgie, Universitätsklinikum Regensburg, Franz-Josef-Strauß-Allee 11, 93053 Regensburg, Germany; 3 Uniklinik Köln, Klinik und Poliklinik für Psychiatrie und Psychotherapie, Kerpener Straße 62, 50937 Köln, Germany

**Keywords:** Colorectal surgery, Cognitive errors, Morbidity & mortality conference, Outcome management, Content analysis

## Abstract

**Background:**

Cognitive errors have a considerable effect on procedural outcome. They play a major role in situational judgement and decision making, especially during cognitively demanding tasks. As such they need to be considered an important factor in medical and surgical procedures. However, whereas cognitive diagnostic errors are well known, as of yet the occurrence of errors due to cognitive heuristics may have been downplayed, underestimated, or simply been ignored during the course of surgical treatment.

**Methods:**

All colorectal resections with anastomosis in 2015 and 2016 (*n* = 230) were prospectively screened for anastomotic failure (*n* = 17/230). During structured Morbidity and Mortality Conferences (MMC) all anastomotic failures were analyzed for both tactical and technical decisions in the pre- and intraoperative setting with potential meaning for the postoperative course, based on the London Protocol. In order to demonstrate the significance of cognitive errors in surgical procedures a structured interview with the individual surgeon was conducted including the video and photo documentation of the individual surgical procedure. The interviews were coded by independent coders who were instructed to identify defined cognitive errors. Inter-coder agreement was calculated using Krippendorff’s alpha.

**Results:**

In 12/17 patients with anastomotic failure after colorectal surgery tactical or technical decisions with potential negative influence on anastomotic healing or the postoperative course were assessed during MMC. In 8/12 procedures a structured interview could be conducted with the operating surgeon. In 7/8 procedures cognitive errors could be identified. In particular we found Anchoring (*n* = 1), Availability Bias (*n* = 1), Commission Bias (*n* = 1), Overconfidence Bias (*n* = 1), Omission Bias (*n* = 2) and Sunk Costs (*n* = 1).

**Conclusion:**

Cognitive errors seem to play an important role during surgical therapy of patients with anastomotic failure after colorectal resection. Consequently, we suggest cognitive errors should attract more interest in research as well as attention in clinical practice.

## Background

Morbidity in colorectal surgery of malignant tumors lies between 30 and 40% [1]. Not least due to their high lethality anastomotic leaks belong to the more feared complications. The basic prerequisite to reducing morbidity is medical error analysis. Concerning anastomotic leak, most patients’ inherent risk factors are the primary focus of medical error analysis [2]. Less often, patient independent factors are investigated as causes of anastomotic leak [3] . In the case of patient-independent factors, noticeable problems in operating technique and strategy, as well as irregularities during processes and structure are to be considered. Where structural quality is more environment-related, noticeable problems in diagnostics, operating technique and strategy, as well as procedural adherence are more individual-related.

In the investigation of individual-related factors of the incidence of adverse events one has to pay close attention to the impact of cognitive errors on the part of the surgeon (physician). In cognitive psychology, the term *cognitive error* is used as a collective term for systematic errors during perception, memory recollection, cognition, and decision making. Usually these errors remain unnoticed and subsequently lead to errors in reasoning and wrong decision making [4–7]. We may assume that an adverse event which cannot be sufficiently explained by patient-related factors, most likely is caused by a cognitive error and a following (unintended) factual error made by the physician or caretaker [8, 9]. This factual error leads to a decision which in turn promotes the adverse event (*cognition-decision-event*) [10] . In the analysis of therapeutic errors primarily diagnostic and medication errors have been of interest [11–13]. Less commonly adverse events are being investigated as a part of surgical procedures, and those studies investigating surgical procedures have been restricted to genuine therapeutic errors such as e.g. the unwanted severance of the bile duct during laparoscopic cholecystectomy [14, 15].

However, improvement in surgical outcome may only be accomplished with extensive and sufficient analysis of adverse events and their causes, including physician-related cognitive errors. As anastomotic leak presents a complication with considerable undesirable consequences, we have conducted this study in order to investigate the occurrence of cognitive errors while performing an anastomosis, subsequent treatment procedure and treatment success of anastomotic leak in colorectal surgery. Importantly, the complications due to ‚genuine’surgical errors such as bile duct injury, are usually directly attributable to the corresponding error. Due to its multifactorial genesis, such a direct causal relation of a cognitive error to decision or event is not so easily established for anastomotic leak. Therefore, our analysis is limited to the occurrence of cognitive errors with regards to the adherence to the established prerequisites for anastomotic healing.

## Material and methods

All procedures performed were in accordance with the ethical standards of the institutional and/or national research committee and with the 1964 Helsinki declaration and its later amendments or comparable ethical standards. The study obtained local ethics committee approval. In order to investigate the occurrence of cognitive errors during the therapy of patients with anastomotic leak in colorectal surgery, we screened colorectal resections performed during the years 2015 and 2016 (not including Hartmann’s procedures and rectal extirpation) for complications. All corresponding patients had passed through a preoperative risk assessment of patient-related risk factors. The approved requirements for healing without complication of the anastomosis (perfusion, absence of tension, absence of fat residues, seam tracking, seam distance) were recorded intraoperatively by video/photo and using a software for standardized method of documentation. All anastomoses and leaks were photo-documented.

**Fig. 1:**
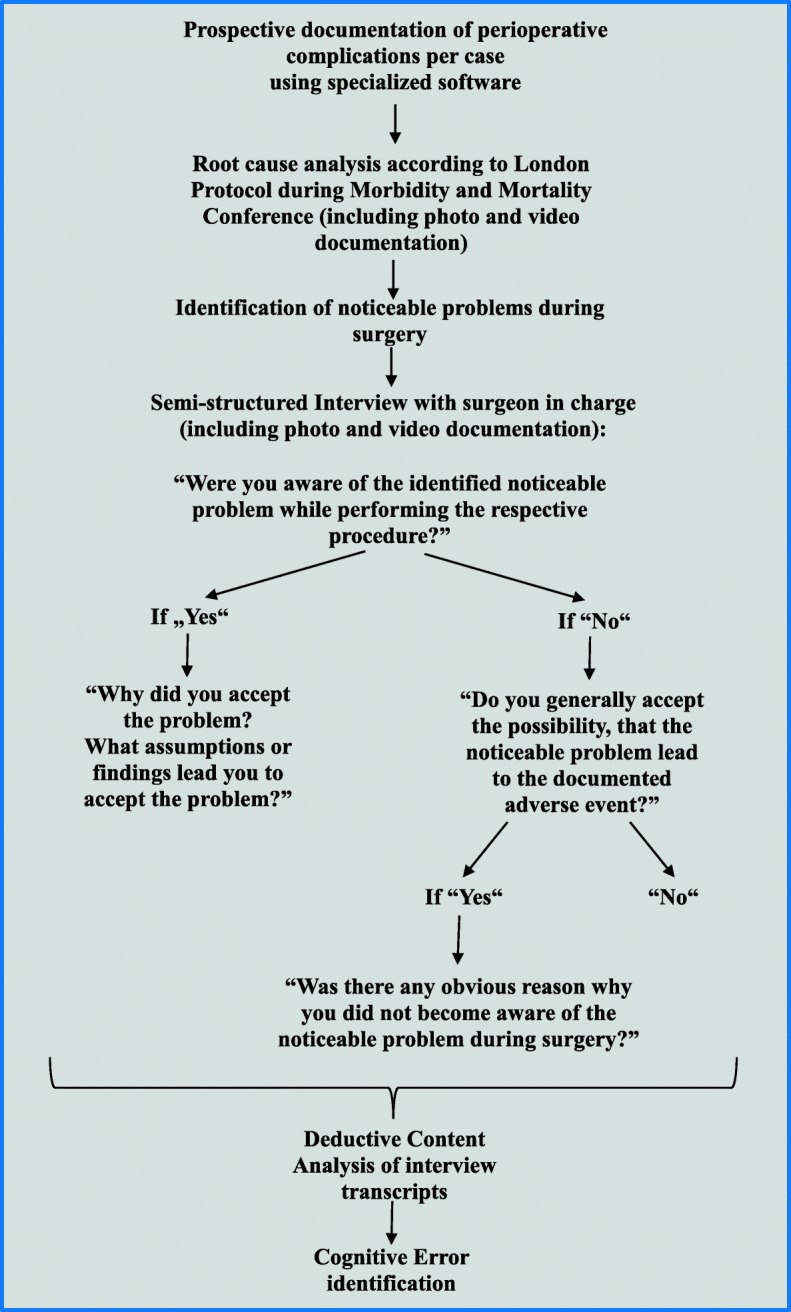
Operational procedure of data acquisition and analysis

The operational procedure for data collection and analysis is depicted in Fig. 1. According to standardized procedure, all complications including anastomotic leak were analyzed in interdisciplinary *morbidity and mortality conferences* (MMC). Anastomotic leak was defined according to Rahbari et al. [16]. The standardized analysis of adverse events during MMC was based on the London Protocol, a standardized instrument of error analysis [17] . Accordingly each adverse event was analyzed with regards to the predetermined parameters: diagnostics, indication, operating technique, operating strategy, follow-up treatment, multidisciplinarity, organization, procedural quality, structural quality, and “other”. Multiple adverse event entries per patient were possible. MMC were conducted weekly and lasted 45 min. They included all practicing surgeons from the departments of General-Abdominal and Minimally Invasive Surgery, the nursing staff management and a representative from the Department of Anesthesiology and Intensive Care at the Hospital Bad Hersfeld, Germany.

Documents of analysis included patient’s record (paper and digital), risk assessment, surgery data analysis and recorded results (using specific analytic software, radiographs (Infinite®; Tampa, FL 33602, USA) as well as video and photo documentation using Storz OR-1® (D-78532 Tuttlingen). The methodological framework for the MMC is depicted in Table 1. All data points (patient-related risk, data recorded during surgery, outcome) including the results from the MMC were documented using software specifically designed for this type of documentation. From this documentation all anastomotic leaks over the period of investigation were identified, including noticeable problems during, before, and after surgery.

**Table 1:** The Morbidity and Mortality Conference as a problem identification tool

Participants	All practicing physicians from Department of Surgery Nursing Management Representative from Department of Anesthesiology
Frequency	weekly
Duration	45 min
Documentation	Patient record (paper und digital) Radiographs Individual Risk Assessment, survey of surgical data and results obtained with specific software Photo and video documentation
Content of Analysis	Individual Risk Assessment, survey of surgical data and results obtained with specific software Photo and video documentation Nursing Documentation Imaging Lab results, Microbiology, Pathology Photo- and Video Documentation

A structured interview was conducted with each attending surgeon whose patient had experienced anastomotic leak due to an identified noticeable problem. Informed consent was obtained from all participating surgeons prior to the interview. The risk for recall bias was countered by the use of video and photo documentation of the noticeably problems potentially causative to the development of the adverse events. Material generation, including interview procedure is depicted in Fig. 1. During the interview, the specific noticeable problems as evaluated and described during MMC were laid out (including surgical record and video/photo documentation). It was then inquired from the surgeon whether he had been aware of the basic requirements for uneventful healing of anastomoses (perfusion, absence of tension, absence of fat residues, seam tracking, seam distance) [18] and of the noticeable problem identified in the MMC during the respective surgical procedure. If the answer to both questions was‚ yes’, the surgeon was asked why he had accepted the noticeable problem or divergence from standard procedure during surgery. If retrospectively the process sequence had contributed to the incidence of anastomotic leak, it was inquired whether its potential impact during surgery (prospectively) had been known to the surgeon and why divergences from protocol had been deemed necessary.

All interviews were documented by the interviewer (PV) and digitalized using MS office Word (Microsoft, Seattle, USA). The interviews were then coded by four independent coders using deductive content analysis [19, 20]. Coders were instructed to identify potential cognitive errors. To this end, coders were provided with consecutively numbered terminology, definitions and illustrations of cognitive errors according to Stiegler et al. [4] (Table 2). For each interview the cognitive error causative to the following adverse event was to be identified. If more than one cognitive error could be identified, coding rules required coders to select the pivotal error. Accordingly, each interview could be assigned one code for a specific cognitive error. Should coders not be able to identify a cognitive error in an interview using the provided explications, they were instructed to code “zero” for “no cognitive error”. All coders were naive to both topic and material, but received explanations of the material generation procedure and interview structure to avoid misunderstanding of questioning. Coders were selected from medical students and physicians not involved in the procedures which preceded material generation in order to guarantee both independence and sufficient knowledge of surgical procedures necessary for coding. Inter-coder agreement was calculated using Krippendorff’s alpha [21].

**Table 2:** Cognitive Errors, Definitions and Illustrations from anesthesiology acc. to Stiegler et al.^4^; Illustrations from surgery as described in this study

Cognitive Error	Definition	Illustration from anesthesiology	Illustrations from surgery
Anchoring	Focusing on one issue at the expense of understanding the whole situation	While troubleshooting an alarm on an infusion pump, you are unaware of sudden surgical bleeding and hypotension	Vascular surgeon focuses on aortic aneurism, but disregards changes indicating carcinoma in the sigma. The aneurism is eliminated first leading to a change in perfusion further adding to the changes in blood supply in the colon. The sigma is excised afterwards under ischemic conditions.
Availibility bias	Choosing a diagnosis because it is in the forefront of your mind due to an emotionally charged memory of a bad experience	Diagnosing simple bronchospasm as anaphylaxis because you once had a case of anaphylaxis that had a very poor outcome	The standard for protecting anastomosis is ileostomy. Therefore an ileostomy was performed although the colon was loaded with feces hindering proper protection of the downstream colon
Premature Closure	Accepting a diagnosis prematurely, failure to consider reasonable differential of possibilities	Assuming that hypotension in a trauma patient is due to bleeding, and missing the pneumothorax	
Feedback Bias	Misinterpretation of no feedback as ‘positive’ feedback	Belief that you have never had a case of unintentional awareness, because you have never received a complaint about it	
Confirmation bias	Seeking or acknowledging only information that confirms the desired or suspected diagnosis	Repeatedly cycling an arterial pressure cuff, changing cuff sizes, and locations, because you ‘do not believe’ the low reading	
Framing effect	Subsequent thinking is swayed by leading aspects of initial presentation	After being told by a colleague, ‘this patient was extremely anxious preoperatively’, you attribute postoperative agitation to her personality rather than low blood sugar	
Commission bias	Tendency toward action rather than inaction. Performing un-indicated manoeuvres, deviating from protocol. May be due to overconfidence, desperation, or pressure from others	‘Better safe than sorry’ insertion of additional unnecessary invasive monitors or access; potentially resulting in a complication	Two specialized surgeons perform a sigma-rectum-resection. The performing surgeon notices fatty residues on the dorsal part of residual rectum (Fig. 2), which the assisting but more experienced surgeon judges to be of no relevance (overconfidence). Due to the resulting pressure the less experienced surgeon, despite his own concerns, made the unindicated decision in favor of an anastomosis, leading to anastomotic leak on the dorsal (fatty) part of the anastomosis.
Overconfidence bias	Inappropriate boldness, not recognizing the need for help, tendency to believe we are infallible	Delay in calling for help when you have trouble intubating, because you are sure you will eventually succeed	The necessary standard of performing perfusion control was not met. An explanation for divergence from protocol was deemed unnecessary
Omission bias	Hesitation to start emergency manoeuvres for fear of being wrong or causing harm, tendency towards inaction	Delay in calling for chest tube placements when you suspect a pneumothorax, because you may be wrong and you will be responsible for that procedure	1. Although indicated to establish absence of tension no severance of the mesocolon out of concern that perfusion possibly might be compromised (Fig. 3) 2. After resection of rectum and bladder a small rigid bladder residue remained, complicating establishing an anastomosis. Instead of Hartmann-resection according to protocol, a primary anastomosis with upstream ileostomy was performed due to fear of worse conditions for later reattachment.
Sunk costs	Unwillingness to let go of a failing diagnosis or decision, especially if much time/resources have already been allocated. Ego may play a role	Having decided that a patient needs an awake fibreoptic intubation, refusing to consider alternative plans despite multiple unsuccessful attempts	After conforming to the standards for checking for the perfusion of the intestinal region responsible for the anastomosis, a small livid discoloration is noticed on the aboral part of the anastomosis. (Fig. 4). Due to the long-lasting surgical procedure in accordance to standards, the discoloration is judged to be of no further concern. A leak develops in this region.
Visceral bias	Counter-transference; our negative or positive feelings about a patient influencing our decisions	Not trouble-shooting an epidural for a laboring patient, because she is ‘high-maintenance’ or a ‘complainer’	
Zebra retreat	Rare diagnosis figures prominently among possibilities, but physician is hesitant to pursue it	Try to ‘explain away’ hypercarbia when MH should be considered	
Unpacking principle	Failure to elicit all relevant information, especially during transfer of care	Omission of key test results, medical history, or surgical event	
Psych-out-error	Medical causes for behavioural problems are missed in favour of psychological diagnosis	Elderly patient in PACU is combative—prescribing restraints instead of considering hypoxia	

In case of diverging codes, the corresponding passages and codes were to be cross-analyzed after inter-coder agreement calculation, until a consensus was reached.

## Results

Over the investigated period, *n* = 230 colorectal surgeries were performed. Morbidity was calculated at *n* = 67/230 (29.1%) and mortality at *n* = 16/230 (6.9%). *N* = 17/230 (7.4%) suffered from anastomotic leak. Mortality from anastomotic leak based on the total of patients operated accounted for 4/230 (1.7%), based on patients with anastomotic leak 4/17 (23.5%).

In 12/17 patients with anastomotic leak noticeable problems could be identified during MMC regarding the basic requirements for uneventful healing of anastomoses or treatment procedure of anastomotic leaks. In 8/12 of these procedures a structured interview could be conducted with the operating surgeon (*n* = 3 surgeons). The remaining 4 procedures had been conducted by a surgeon who had left the department prior to the start of this investigation. All surgeons interviewed were considered high volume surgeons according to the definition by Archampong et al. [22]. 1/8 interviews had to be excluded from the analysis, as the noticeable problem could not be evaluated consistently, even under inclusion of the video and photo documentation. In 7/8 procedures cognitive effects could be delineated using content analysis. Calculation of Krippendorff’s alpha yielded a strong inter-rater agreement of α = 0.83. In two cases diverging codes were identified. After the most applicable cognitive error had been identified for these cases, the observable errors included: anchoring, availability bias, commission bias, omission bias (*n* = 2), sunk costs, and overconfidence. Table 2 gives an overview as well as the definition of the cognitive errors according to Stiegler et al. [4] used herein.

In some cases external factors or technical factors added to the development of cognitive errors. In one case, multidisciplinary specialization in double affection (abdominal aortic aneurysm and sigmoid colon carcinoma), in two other cases a process standardization, and in one other case technical factors (minimally invasive procedure) seemed of importance.

## Discussion

Outcome quality and patient safety are of great importance in surgery. They are determined by the incidence of adverse events which is referred to as *morbidity*. In the elective surgery of colon and rectum, morbidity lies between 30 and 40% [1, 23]. Among adverse events, anastomotic leak is of special significance due to its high risk of mortality.

As of yet, the importance of cognitive errors during the treatment course of patients with adverse events has not been investigated sufficiently in health care. In this study we were interested in the incidence of cognitive errors during the pre-, intra-, and postoperative course of patients with anastomotic leak after colorectal surgery. Because anastomotic failure is multifactorial we focused on the adherence to the accepted prerequisites for anastomotic leak [18]. We analyzed structured interviews using deductive content analysis [19, 20].

**Fig. 2:**
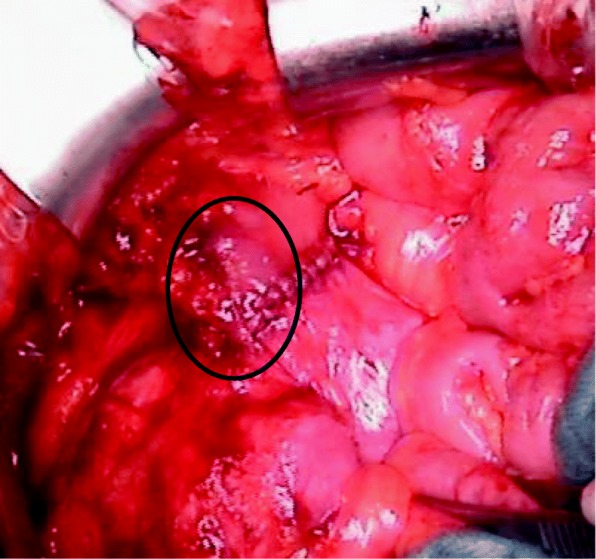
Picture from in-surgery documentation: livid discoloration (circled) at site of anastomosis

**Fig. 3:**
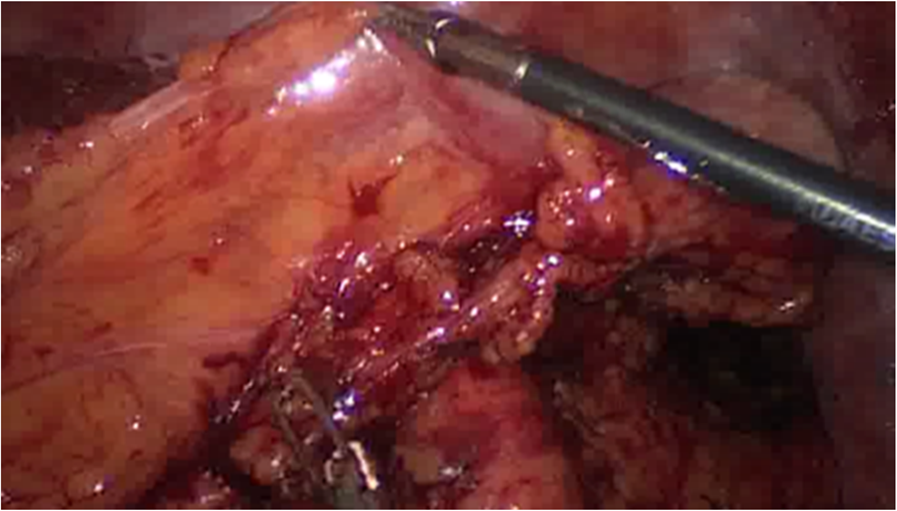
Picture from in-surgery documentation: tense mesocolon

**Fig. 4:**
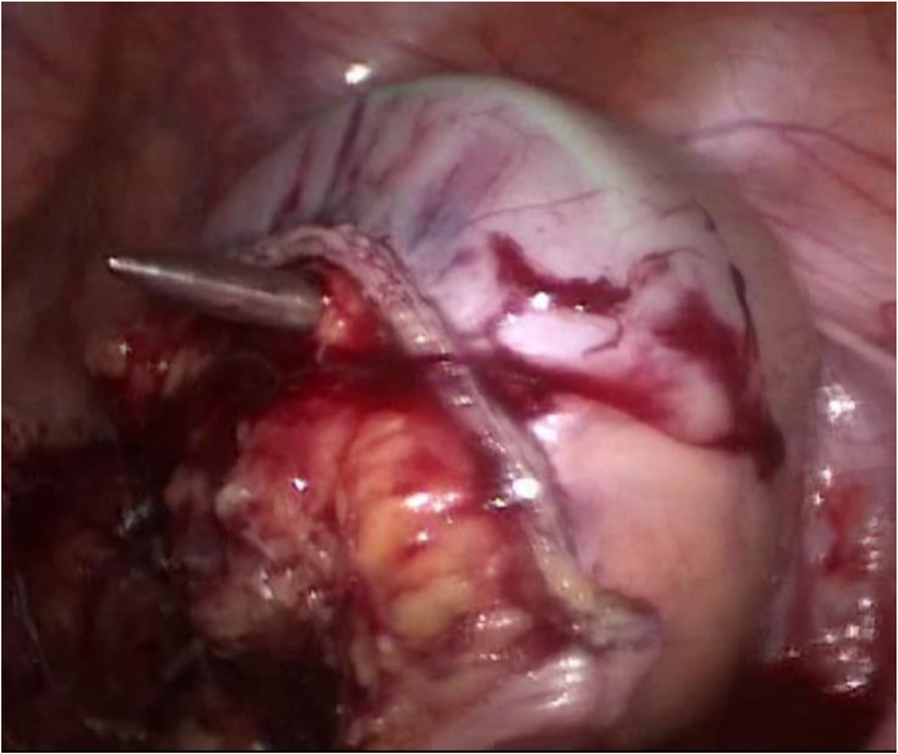
Picture from in-surgery documentation: rectum with fatty residues

We were able to detect cognitive errors in 7 of 8 cases with anastomotic failure investigated so that cognitive errors may be much more important than assumed. In these seven cases, cognitive errors occurred both pre-operatively and intra-operatively. Pre-operatively they affected diagnostics and tactical decisions. Intra-operatively they affected technical (adherence to the established prerequisites for anastomotic healing) and tactical decisions (performance of anus praeter). We identified Anchoring (*n* = 1), Availability Bias (*n* = 1), Commission Bias (*n* = 1), Overconfidence Bias (*n* = 1), Omission Bias (*n* = 2) and Sunk Costs (*n* = 2) (Table 2). The accepted prerequisite for anastomotic healing most frequently affected was perfusion (*n* = 3; Fig. 2), tension (*n* = 1; Fig. 3), fatty residues on the dorsal part of residual rectum (*n* = 1; Fig. 4) and tactical decisions concerning the performance of the stoma, which subsequently negatively affected the treatment course after anastomotic leak (*n* = 2).

Besides individual causes [4] internal (surgery-related) factors or external (systemic) factors may contribute to cognitive errors. Concerning the cases presented in this study, internal factors were present in the form of the complexity of surgical procedures (including multidisciplinarity, and the development of surgical technique). An external factor may be the increasing economization in the field of surgery and health care in general.

### Cognition and Internal Factors - Complexity in the Field of Surgery

The majority of cognitive errors identified in our material was either directly or indirectly related to the complexity of surgical procedures. The complexity of a task increases its error-proneness [12, 15]. Accordingly, aspects and developments which increase complexity, as well as the subsequent attempts to reduce complexity will have to be considered for the field of surgery. Attempts to reduce complexity are the implementation of standards [14] and specialization [24].

#### Standards

Cognitive errors demonstrated in this study lead to decisions diverging from accepted prerequisites of anastomotic healing as standard procedures. Standards limit individual flexibility of interpretation and action through the implementation of specific procedures and their control (e.g. photo and video documentation) [14]. They may not always agree with the individual approach preferred by some health care professionals. Nevertheless, surgery is often determined by complex situations which increase the risk of adverse events [12, 15]. Standards therefore improve patient safety and non-adherence or deviation from standards poses a well-known safety risk [25].

Naturally divergences from standard are not undertaken deliberately under the assumption of adverse events. Rather, it may be assumed, that an error causes a pseudo-rationalization which makes a divergence without assumption of risk appear feasible. Overconfidence defined as the overestimation of the own ability to judge may be crucial (Table 2).

The assessment of the importance of divergence from standards relevant to safety is impeded by a variety of factors. Pseudo-rationalization causes the divergence to go virtually unnoticed and hence errors mostly remain unevaluated. With the interview and retrospective analysis of video and photo documentation we were able to uncover and prospectively avoid this problem in our study.

The recognition of the relevance of divergences from standardized procedures to patient safety is further limited as cognitive errors do not regularly cause adverse events. Accordingly, a study observing the behavior causative to errors in decision making exhibited by pilots, found that a divergence from procedure was recorded in 55%, however, only 3% of these divergences lead to an adverse event [26]. The question to which extent such divergences from predetermined procedure are relevant to surgery, cannot be answered by this study as we refrained from examining all colorectal resections, but focused on those which resulted in anastomotic leak. In this context however, Amalberti et al. [27] emphasize that a lack of quantitative data should not mask the relevance of a problem. The authors note that in health care significantly less procedural regulations are in place than in other high-risk areas. As procedural regulations structure complex procedures and improve cognitive controllability, the implementation of standardized procedures especially in the complex tasks common in surgery are necessary to reduce cognitive errors. This might not only improve patient safety but also simplify surgical training.

In this study violation of rules and adverse events concerned surgeons with high volume criteria. We conclude that cognitive errors are of concern to every surgeon independent from experience. This conclusion confirms results obtained by Choudry et al. [28]. The authors conducted an analysis of articles (*n* = 62) concerning the connection between experience and quality criteria in health care. They found that in 73% of articles analyzed, a negative relationship between experience and adherence to criteria could be observed. The aversion to the implementation of standards was especially high in experienced surgeons [28].

Importantly, standardization should not be applied uncritically. Apart from the mentioned benefits, standardization bears the risk of an *availability bias*, meaning that with practice the predetermined standard is cognitively most available and hence performed regardless of proper indication. In our analysis this became apparent twice with the standard “protective ileostomy”. In one case this standard procedure was implemented although the downstream colon was insufficiently decompressed and reachable via colostomy. In another case the colon was loaded with feces and ileostomy could not provide the necessary relief for the anastomosis. In order to preserve the advantages of standards (e.g. decrease in complexity) and at the same time avoid disadvantages (e.g. availability bias), a sufficient differentiation of standards is necessary. Furthermore, standards do not excuse from critical thinking in an individual case.

#### Specialization

Error proneness during complex procedures may be reduced by increasing specialization and is accepted as a key element of improving outcome. However, specialization may lead to cognitive errors. In one of our analyzed cases, it promoted *anchoring*, which contributed to a surgical sequence which retrospectively should have been planned differently. The corresponding patient was initially admitted with an abdominal aortic aneurism. After the elimination of the aneurism the patient developed regional ischemia of the colon, compelling resection. This resulted in the diagnosis of sigmoid colon carcinoma. Evidence of the tumor mass had already been visible in the CT- image conducted for sizing of the aortic stent, but had been overlooked due to the specialized focus on the aneurism. This finding retrospectively could be established in the CT-image conducted for sizing of the aortic stent. Retrospectively speaking, since perfusion is of crucial importance for anastomotic healing [18], a different sequence of surgical procedures with primary oncological resection of the sigma under unaltered conditions of perfusion and secondary elimination of the aortic aneurism could have prevented anastomotic leak.

#### Surgical technique

Lastly, complex technical aspects such as the application of minimally invasive surgical methods may pose a special cognitive demand. They force the surgeon to specific safety considerations [29]. In our interviews limited haptics seemed to pose a particular difficulty. Thus, one surgeon indicated in the respective interview that he refrained from severing the mesocolon as indicated to establish an absence of tension in the anastomosis because due to a lack of haptic control he feared to endanger vessels allegedly located in this area. He assessed the blood supply to be sufficient, wherefore, a switch to an open procedure was foregone. Nine months later a resection which had become necessary due to anastomotic leak and subsequent stenosis showed that the vessels were not located in the suspected area.

### Cognition and external changes

Other than internal surgery-specific factors, external, systemic factors may lead to cognitive errors. In principal, indicators of productivity transfer economic pressure and hence may influence quality of health care in a negative way [30]. Indicators of productivity exert influence on cognition through sunk costs, which were also detected in this study. Sunk costs leads to an overoptimistic judgement in a given situation [7, 31]. Therefore, sunk cost do not necessarily reflect personal economic gain or loss, but e.g. the goal to avoid additional surgical intervention by increasing operating time may lead to cognitive errors which in turn may negatively influence decision making. Every surgeon should be trained to resist this pressure.

Awareness of cognitive errors may be improved with metacognitive training which provides surgeons with techniques to identify vulnerability to biases. It has been criticized that a direct evidence of connection between metacognitive training and improving medical procedures was lacking, was primarily theoretical in nature, and was not convertible to health care [32]. However, newer studies have demonstrated the positive effect of cognitive training on the performance of surgeons [33]. The critique may further be refuted by the results from this study as well as studies on the influence of cognition in laparoscopic cholecystectomy [13, 14]. Adverse events are usually multi-attributable which also rules true for cognitive errors. Their uncovering and reduction may therefore lead to the reduction of adverse events and thereby promote patient safety. We argue that the metacognitive analysis of cognitive errors should become an integral part of MMC.

## Limitations

This study has several limitations. The interviews were conducted retrospectively and in some cases were not carried out promptly after surgery or after the respective MMC. This potential recall bias was countered by the use of video and photo documentation of the surgical procedures. Not all surgeons who operated on the selected sample could be included in the interviews. Together with a relatively low incidence of anastomotic leak the sample size for analysis must remain small and we do not consider our material saturated [34, 35]. However, the primary goal of this study was not the detection of all cognitive errors possibly contributing to the incidence of anastomotic leak. More appropriately we wish to raise awareness to the fact, that cognitive errors are a major and largely neglected risk factor in health care and surgery, and aim to encourage both scientific and clinical discussion of cognitive biases as causes of medical complications.

## Conclusion

Whereas to date the influence of patient-related risk factors on anastomotic leak has been the primary target of research, the cognitive errors of the physician in charge were at the heart of investigation in this study. These errors were not studied experimentally or with regards to genuine therapeutic errors, but in the everyday environment of the clinical practice of colorectal surgery and its specific complications. Cognitive errors seem to play an important role during surgical therapy of patients with anastomotic failure after colorectal resection. Consequently, we suggest cognitive errors should attract more interest in research as well as attention in clinical practice. We suggest that the implementation of metacognitive training in surgical training could significantly improve patient safety.

## Abbreviations

MMC: Morbidity and mortality conference(s)
